# Relationship between serum prealbumin level and prognosis of community-acquired bacterial meningitis in adults: a retrospective cohort study

**DOI:** 10.3389/fneur.2024.1424069

**Published:** 2024-09-16

**Authors:** Jing Shang, Lanping Xue, Hongping Zhao, Xiaopeng Cui, Lijuan Shangguan, Hailong Wang, Xinyi Li

**Affiliations:** ^1^Department of Neurology, Third Hospital of Shanxi Medical University, Shanxi Bethune Hospital, Shanxi Academy of Medical Sciences, Tongji Shanxi Hospital, Taiyuan, China; ^2^Department of Neurology, Shanxi Bethune Hospital, Shanxi Academy of Medical Sciences, Tongji Shanxi Hospital, Third Hospital of Shanxi Medical University, Taiyuan, China; ^3^Department of Hepatobiliary and Spleen Tumor Surgery, Shanxi Provincial People’s Hospital, Taiyuan, China

**Keywords:** community-acquired bacterial meningitis, CABM, prealbumin, PA, retrospective cohort study

## Abstract

**Background:**

Low serum prealbumin levels have been identified as a predictor of infectious complication in critically ill patients. However, the association in patients with Community-acquired bacterial meningitis (CABM) remains unclear. The aim of this study is to investigate the relationship of prealbumin and the poor outcome of CABM through a retrospective cohort study.

**Methods:**

A total of 77 patients of CABM were enrolled. They were divided into good outcome group (GOS: 5) and a bad outcome group (GOS: 1–4). Serum prealbumin and other clinical records were measured within 24 h after admission.

**Results:**

Among the included patients, 38(65.52%) had a bad outcome (the GOS score between 1 and 4). The mean age of the overall cohort was 45.3 ± 15.9 years, and 58.6% of patients were male. The mean prealbumin level in the bad outcome group was 115.4 ± 49.4 mmol/L, while the mean level in the good outcome group was 199.1 ± 49.3 mmol/L (*p* < 0.001). Individuals with plasma prealbumin level ≤180 mmol/L had a 3.32-fold higher risk of CABM than those with normal plasma prealbumin level [OR = 4.32 (1.02 ~ 18.24), *p* < 0.05].

**Conclusion:**

Reduced plasma prealbumin level is independently associated with the poor outcome of CABM. Plasma prealbumin level might help to identify patients at high risk of bad outcome.

## Introduction

Community-acquired bacterial meningitis (CABM) is an impactful disease with substantial mortality and morbidity worldwide (1). Nowadays, the common pathogenic bacteria of CABM include *Streptococcus pneumoniae*, *Neisseria meningitidis*, and *Listeria monocytogenes*. The annual incidence was about 0.6–4 cases per 100,000 adults. Despite significant improvements in medical care, CABM still has a high risk of death or serious sequelae (2–5). Therefore, we need to look for prognostic related factors to evaluate poor outcome of this disease.

Some lab test indexes already be used to predict prognosis of infectious diseases. Chief among them are some indicators of inflammation, such as C-reactive protein, lactate dehydrogenase, interleukin, D-dimer, and procalcitonin. Serum Prealbumin is also considered be associated with infectious diseases.

Prealbumin, also named transthyretin, is a 54-kDa protein. It is mainly synthesized by liver and choroid plexus. Prealbumin participates in the transport of thyroxin and retinol. It is a negative acute-phase reactant. It is decreased in inflammation, liver disease, malnutrition and dyscrasia (6). Some studies have shown that reduced prealbumin level was associated with inflammatory responses.

The aim of this study is to investigate the predictive value of prealbumin for the poor outcome of CABM through a retrospective cohort study.

## Methods

### Design and population

This retrospective cohort study aimed to estimate the potential association between prealbumin level and the severity of bacterial meningitis. It was performed in accordance with the Strengthening the Reporting of Observational studies in Epidemiology (STROBE) statement, and the Declaration of Helsinki, and was approved by the institutional review board of ShanXi Bethune Hospital.

Between November 2014 and June 2023, 77 consecutive patients who met all the diagnostic ceiteria of bacterial meningitis were included, that is: (1) age ≥14 years; (2) acute onset of illness; (3) CSF bacterial cultures or PCR were positive or at least one specific cerebrospinal fluid finding predictive of bacterial meningitis (according to the criteria of Spanos and colleagues): CSF glucose concentration < 1.9 mmol/L, CSF to blood glucose ratio < 0.23, CSF protein concentration > 2.2 g/L, CSF leucocyte count >2,000/μL, or CSF neutrophil count >1,180 μL. (4) With or Without: stiff neck, headache, seizure or altered mental status (7).

Study exclusion criteria include: (1) surviving patients with a hospital stay of fewer than 3 days; (2) positive human immunodefificiency virus status, meningitis without clinical brain involvement, brain abscess, prion diseases, cerebral malaria, and noninfectious CNS diseases (8); (3) patients with head trauma or neurosurgery in the previous month, or those with a neurosurgical device or missing outcome (9).

### Patient data

Clinical and laboratory data were obtained from the hospital records. Fever was defined as a temperature above 37.5°C during the course of the disease.

Outcome was graded according to the Glasgow Outcome Scale (GOS) score at discharge (10): a score of 1 corresponds to death, 2 to a vegetative state (unable to interact with the environment), 3 to severe sequelae and dependency upon others in daily life, 4 to moderate sequelae but with the ability to live independently, and 5 to no or only minor sequelae. A favorable outcome was defined as a score of 5 and an unfavorable outcome as a score of 1–4.

### Statistical analysis

All the analyses were performed with the statistical software packages R (The R Foundation)^1^ and Free Statistics software versions 1.7. Descriptive statistics [mean ± standard deviation and frequency (percentage)] are used to report baseline cohort characteristics. All significance tests were two-sided, and a value of *p* < 0.05 was considered statistically significant.

There were no missing data on demographic characteristics and clinical examinations. Categorical variables were presented as frequency and percentage, continuous variables as mean ± standard deviation. Distributions of clinical characteristics were compared between groups using the Mann–Whitney test for continuous variables and the chi-squared test for categorical variables.

We used Logistic regression model to investigate the association between serum prealbumin level and the outcome, adjusting for confounding factors, including age, sex, T, R, SBP, base illness, diabetes, fever, headache, mental behavior disorder, Meningeal irritation sign, epileptiform seizure, paralysis, GCS, ICU, image abnormal, cfp, WBC, NEUT, NEUT%, CRP, PCT, TP, ALB, BUN, PA, K, Na, Cl, P, Ca, D-dimer, CSF wbc, CSF absolute value of mononuclear cells, CSF absolute value of multiple nuclear cells, csf RBC, csf pro, csf Glu, CSF cl, Cerebrospinal fluid culture positive, NGS positive, and adrenoglucohormone treatment. The variables with a *p* < 0.1 were included in the multivariable analysis.

Multivariable conditional logistic regression models were used to assess the association between prealbumin and the outcome. Prealbumin also was used as a categorical variable to assess its association with the severity of illness by multivariable conditional logistic regression models. Unadjusted and adjusted conditional odds ratios with 95% confidence intervals (CIs) were calculated. Three adjusted logistic regression models were used. Model I was adjusted for sex and age. Model II was adjusted for sex+age + Ca + ALB+D-dimer, Model III was adjusted for sex+age + Ca + ALB+D-dimer+csf pro+csf Glu + CSF absolute value of multiple nuclear cells and adrenoglucohormone treatment.

Despite the relatively small sample size of bacterial meningitis patients, stratified analyses were conducted to assess the association of elevated prealbumin level and the outcome by subgroup.

## Results

We enrolled 77 patients with bacterial meningitis. Of these 77 patients, 39 were bad outcome. The baseline characteristics of 77 patients are shown in Table 1. Among the included patients, those with a bad outcome (GOS score between 1 and 4) numbered 38 (65.52%). The mean age of the overall cohort was 45.3 ± 15.9 years, and 58.6% of patients were male.

**Table 1:** Characteristics of patients included in the analysis (*n* = 77).

Variables	Patients with poor outcome (*n* = 39)	Patients with nonpoor outcome (*n* = 38)	*p*
Sex (males, %)	23 (59)	23 (60.5)	0.89
Age (years)	49.4 ± 17.8	45.1 ± 15.4	0.255
T (°C)	37.6 ± 1.0	37.9 ± 1.2	0.159
R	23.7 ± 8.3	23.2 ± 7.6	0.779
SBP (mmHg)	134.8 ± 20.1	125.3 ± 21.1	0.046
Base illness (%)	32 (82.1)	27 (71.1)	0.254
Diabetes (%)	7 (17.9)	3 (7.9)	0.31
Fever (%)	36 (92.3)	37 (97.4)	0.615
Headache (%)	29 (74.4)	28 (73.7)	0.946
Mental behavior disorder (%)	20 (51.3)	7 (18.4)	0.003
Meningeal irritation sign (%)	29 (74.4)	29 (76.3)	0.842
Epileptiform seizure (%)	8 (20.5)	7 (18.4)	0.817
Paralysis (%)	7 (17.9)	2 (5.3)	0.154
GCS	10.1 ± 3.8	12.4 ± 2.8	0.003
ICU (%)	29 (74.4)	19 (50)	0.027
Image abnormal (%)	25 (65.8)	33 (86.8)	0.031
Cfp (mmH_2_O)	232.6 ± 91.8	230.7 ± 79.0	0.929
WBC (*10^9^/L)	16.7 ± 9.6	17.6 ± 8.4	0.663
NEUT (*10^9^/L)	82.1 ± 10.8	87.4 ± 6.1	0.01
NEUT% (*10^9^/L)	14.1 ± 8.2	14.8 ± 7.3	0.69
CRP (mg/L)	112.4 ± 75.8	107.4 ± 82.1	0.053
PCT (ng/mL)	9.2 ± 14.4	7.0 ± 7.3	0.481
TP (mmol/L)	64.6 ± 8.6	67.4 ± 7.2	0.188
ALB (mmol/L)	34.4 ± 6.0	40.1 ± 4.4	< 0.001
PA (mmol/L)	115.4 ± 49.4	199.1 ± 49.3	< 0.001
K (mmol/L)	3.5 ± 0.5	3.6 ± 0.5	0.801
Na (mmol/L)	133.1 ± 5.9	135.9 ± 4.2	0.018
Cl (mmol/L)	100.8 ± 6.0	101.8 ± 4.9	0.42
P (mmol/L)	0.8 ± 0.3	0.9 ± 0.3	0.057
Ca (mmol/L)	2.1 ± 0.2	2.2 ± 0.1	0.002
D-dimer (μg/L)	1590.8 ± 1678.7	697.6 ± 698.2	0.004
CSF wbc (*10^6^/L)	2357.8 ± 4266.8	4959.1 ± 11955.7	0.205
CSF monomorphonuclear cells (*10^6^/L)	523.1 ± 930.4	918.8 ± 1819.5	0.231
CSF polymorphonuclear cells (*10^6^/L)	1834.8 ± 3411.5	4149.0 ± 10326.4	0.189
Csf RBC (*10^6^/L)	7007.6 ± 27110.3	7968.4 ± 45976.9	0.911
Csf pro (g/L)	3.1 ± 1.6	2.5 ± 1.6	0.102
Csf glu (mmol/L)	1.4 ± 1.3	2.0 ± 1.6	0.053
CSF cl (mmol/L)	115.2 ± 6.6	118.6 ± 7.1	0.03
Cerebrospinal fluid culture positive (%)	14 (35.9)	5 (13.2)	0.021
NGS positive (%)	10 (26.3)	12 (32.4)	0.561
Adrenoglucohormone treatment (%)	30 (76.9)	23 (60.5)	0.12

T, temperature; R, respiratory; SBP, systolicbloodpressure; GCS, Glasgow Coma Scale; CFP, Cerebrospinal fluid pressure; NEUT, neutrophilic granulocyte; PCT, Procalcitonin; TP, Total Protein; ALB, albumin; PA, prealbumin; CSF, cerebrospinal fluid; NGS, Next Generation Sequencing.

Patients with a bad outcome versus those with good outcome had significantly higher values in SBP, Mental behavior disorder, GCS, image, NEUT, ALB, FA, BUN, Na, Ca, D-dimer (*p* < 0.05). The mean prealbumin level in the bad outcome group was 115.4 ± 49.4 mmol/L, compared to 199.1 ± 49.3 mmol/L in the good outcome group (*p* < 0.001). Whereas other parameters studied were similarly distributed.

Decreased serum prealbumin levels, that is, <180 mmol/L, were detected in 82.1% of patients with a bad outcome and in 36.8% of patients with a good outcome. The relationship between serum prealbumin level and outcome is presented in Table 2. Univariate conditional logistic analysis showed a concentration-dependent association between serum prealbumin level and the outcome of bacterial meningitis (OR 1.01, 95%CI 0.95 ~ 0.98, *p* < 0.001). When serum prealbumin level was included as a categorical variable, the association with the outcome of CABM [OR, 7.84, 95% CI, (2.74 ~ 22.4)] was significant. Adjustment did not significantly change the results [OR, 8.87; 95% CI, (2.89 ~ 27.28)] for model I, [OR, 4.2; 95% CI, (1.07 ~ 16.44)] for model II, and [OR, 4.32; 95% CI, (1.02 ~ 18.24)] for model III.

**Table 2:** Association between serum prealbumin level and the outcome of CABM in multiple regression model.

Variable	No. of study participants (%)		Crude		Adjusted mode l^a^		Adjusted model II^b^		Adjusted model III^c^	
	n.total	Bad outcomes	OR (95%CI)	*P*	OR (95%CI)	*P*	OR (95%CI)	*P*	OR (95%CI)	*P*
PA > 180 mmol/L	31	7 (22.6)	1(Ref)		1(Ref)		1(Ref)		1(Ref)	
PA ≤180 (mmol/L)	46	32 (69.6)	7.84 (2.74 ~ 22.4)	<0.001	8.87 (2.89 ~ 27.28)	<0.001	4.2 (1.07 ~ 16.44)	0.039	4.32 (1.02 ~ 18.24)	0.047

a Model I adjusted for sex, age.

b Model II adjusted for sex, age, Ca, ALB, D-dimer.

c Model III adjusted for sex, age, Ca, ALB, D-dimer, csf pro, csf glu, CSF polymorphonuclear cells, Adrenoglucohormone treatment.

OR, odd ratio; CI, confidence interval. ALB, albumin; PA, prealbumin.

The results of interaction analyses of the association between decreased serum prealbumin levels and the bad outcome of bacterial meningitis in subgroups are presented in Table 3. Despite the limited sample size in some subgroups, the association between serum prealbumin levels and the outcome of bacterial meningitis in the stratified analyses was consistent with that in the multivariable logistic analysis. There was no significant difference in the strength of the association between the stratified subgroups, which means no interactive role was revealed in the association between decreased serum prealbumin levels and the bad outcome of bacterial meningitis.

**Table 3:** Association between serum prealbumin level and the outcome of community-acquired bacterial meningitis in subgroups.

	Patients with poor outcome (*n* = 39)		Patients with nonpoor outcome (*n* = 38)		Mutually adjusted OR (95%CI)^a^	*P*-value for interaction
Subgroup	PA ≤180 mmol/L	PA > 180 mmol/L	PA ≤180 mmol/L	PA > 180 mmol/L		
Age, years						0.235
≤60	20	6	9	20	1.88 (0.23, 15.41)	
>60	12	1	5	4	Inf (0, Inf)	
Sex						0.923
Male	18	5	5	18	10.98 (0.58, 207.48)	
Female	14	2	9	6	279558.64 (0.02, 3518730069979.74)	
Image abnormal						0.592
Yes	21	4	12	21	5.49 (0.96, 31.41)	
No	10	3	2	3	Inf (0, Inf)	
Adrenoglucohormone treatment						0.066
Yes	27	3	7	16	129.18 (2.99, 5574.76)	
No	5	4	7	8	0.19 (0, 13.76)	
Cerebrospinal fluid culture						0.187
Positive	12	2	3	2	523.83 (0, Inf)	
Negative	20	5	11	22	4.88 (0.94, 25.38)	
NGS						0.235
Positive	5	2	6	6	5.32 (0, 270358.81)	
Negative	23	5	8	17	7.85 (0.97, 63.7)	

PA, prealbumin; NGS, Next Generation Sequencing.

a Each stratification adjusted for all the factors (sex, age, Ca, ALB, D-dimer, csf pro, csf Glu, polymorphonuclear cells, adrenoglucohormone treatment) except the stratification factor itself.

## Discussion

Our research is the initial cohort study examining the correlation between plasma prealbumin concentration and CABM outcome. Our key finding suggests a correlation between plasma prealbumin levels and poor outcome associated with CABM. This relationship remained strong in multivariate conditional logistic regression model, where plasma prealbumin concentration was considered both as a continuous and categorical variable. Those with plasma prealbumin levels below or equal to 180 mmol/L exhibited a 3.32-fold increase in CABM risk compared to individuals with normal plasma prealbumin levels.

Bacterial meningitis can be quickly fatal and may causes severe disability in the survivors. Survivors might have some complications, such as cognitive impairment, mental and behavioral abnormal condition, hearing loss, motor weakness, paralysis, or epilepsy; although there was few data about low-resource settings, one study found that about a quarter of survivors had neuropsychological sequelaes 3–60 months after discharge (11, 12).Therefore, bacterial meningitis places significant burden on families, communities and society (13, 14).

Previous researches have identified various factors that can predict poor outcomes in bacterial meningitis, including reduced level of consciousness at admission, cranial nerve palsy, low white cell count in cerebrospinal fluid, and specific elements linked to pneumococcal infection (venerable age; otitis or sinusitis, pneumonia, or immunocom promise) or systemic compromise(cardiac acceleration and Increased C-reactive protein) (2, 9, 15, 16). Prealbumin, considered a negative acute-phase reactant, may help doctors to identify the risk of poor outcome.

Serum prealbumin is a plasma protein that synthesized by hepatocytes, its biological half-life is very short (1.9 days). It plays an important role in transporting thyroxine and vitamin A, as well as enhancing the immunity by promoting lymphocyte maturation. Both serum prealbumin and albumin can be used as important indicator to monitor nutritional status and nutritional support. But prealbumin’s half-life is shorter, it is more sensitive than serum albumin (17, 18).

Serum prealbumin is associated with some infectious diseases. For example, in cases of COVID-19, lower prealbumin levels are often indicative of a heightened risk of complications and mortality (19). Patients diagnosed with tuberculosis (TB) frequently exhibit decreased prealbumin levels, and a low level of prealbumin can forecast poorer outcomes, including treatment failure and relapse (20). Sepsis, caused by a dysregulated host response to infection, presents another condition where prealbumin levels can act as a prognostic indicator. In septic patients, lower prealbumin levels are correlated with increased morbidity and mortality (21).

The mechanism linking serum prealbumin levels to infectious outcome is not fully understood. It is believed that inflammatory mediators may damage hepatocytes and hepatic sinusoidal endothelial cells, causing ischemia, hypoxia, and fibrotic liver tissues. This damage may result in reduced prealbumin synthesis and subsequently lower serum albumin levels (17, 22, 23). Circulating albumin has been shown to interact with inflammatory mediators to enhance neutrophil activation and increase phagocytosis. Recent research indicates reductions in serum albumin levels among critically ill patients may primarily linked to inflammation rather than underlying nutritional deficiencies (24).This suggests that the relationship between albumin and inflammatory agents plays a crucial role in determining infectious outcomes (25, 26).Some studies have pointed to the interaction between circulating albumin and inflammatory mediators, which can facilitate processes such as neutrophil degranulation and enhanced phagocytosis. This intricate interplay highlights the complexity of the immune response in critically ill patients and underscores the importance of understanding the influence of inflammatory mediators on albumin levels in such contexts (27, 28).

However, there are several limitations of this study: the sample size was relatively small, treatment protocols were not standardized, and lake long-term of prognosis and long-term mortality; As a retrospective cohort study, detailed socioeconomic data such as BMI, food and housing security, insurance status, and occupational status were not consistently documented in the records and are thus unavailable for our analysis, it may cause the consequent limitations on the availability of certain data points.

## Conclusion

In summary, a decrease in plasma prealbumin levels is independently linked to adverse outcome in CABM patients. Assessing plasma prealbumin levels could potentially help identify patients at higher risk for poor outcome. However, a randomized trial is necessary to confirm the potential causal relationship between reduced plasma prealbumin level and CABM outcome.

## Data Availability

The raw data supporting the conclusions of this article will be made available by the authors, without undue reservation.

## Footnotes

^1^http://www.R-project.org
